# Spongiose

**Published:** 1868-04-01

**Authors:** 


Spongiose. — The introduction of prepared sponge into common use, in the construction of mattrasses, cushions, etc., it is to be hoped will go far toward counteracting the deplorable effects of certain practices, which, according to Prof. H. R. Storer, threatens the future magnitude of the census. Athenæus says it was the ancient practice to put sponges into beds, as incentives to venery. Paulus Ægineta mentions it among the list of aphrodisiacs. The vulgar saying, "Poor men for children," has in it the germ of a profound physiological truth. Infertility is one of the penalties of luxurious living, and absence of children among the wealthy and fashionable, is by no means an evidence of criminal methods of prevention.

